# Evaluation of Strategies for Reducing Vancomycin-Piperacillin/Tazobactam Incompatibility

**DOI:** 10.3390/pharmaceutics15082069

**Published:** 2023-08-01

**Authors:** Anthony Martin Mena, Laura Négrier, Anthony Treizebré, Marie Guilbert, Lucille Bonnaire, Valentine Daniau, Gabie Leba Bonki, Pascal Odou, Stéphanie Genay, Bertrand Décaudin

**Affiliations:** 1 Univ. Lille, CHU Lille, ULR 7365—GRITA—Groupe de Recherche sur les formes Injectables et les Technologies Associées, F-59000 Lille, France; laura.negrier@univ-lille.fr (L.N.); lucille.bonnaire@gmail.com (L.B.); valentine.daniau@gmail.com (V.D.); gabie.lebabonki.etu@univ-lille.fr (G.L.B.); pascal.odou@univ-lille.fr (P.O.); stephanie.genay@univ-lille.fr (S.G.); bertrand.decaudin@univ-lille.fr (B.D.); 2 Univ. Lille, CNRS, Centrale Lille, Univ. Polytechnique Hauts-de-France, UMR 8520—IEMN—Institut d’Electronique de Microélectronique et de Nanotechnologie, F-59000 Lille, France; anthony.treizebre@univ-lille.fr (A.T.); marie.guilbert@univ-lille.fr (M.G.)

**Keywords:** vancomycin, piperacillin/tazobactam, drug incompatibility, infusion, particulate load, in-line filter

## Abstract

Background: Drug incompatibility is defined as a physical-chemical reaction between two or more injectable drugs and that results mainly in precipitation or insolubility. Several strategies for reducing incompatibilities have been implemented empirically in intensive care units. However, these strategies have never been compared directly (and particularly in terms of the particulate load and drug mass flow rate) under standardized conditions. The objective of the present in vitro study was to evaluate the impact of various strategies for preventing incompatibility between simultaneously infused vancomycin and piperacillin/tazobactam. Methods: An in-line filter, a dilute vancomycin solution (5 mg/mL), and an alternative saline administration line were evaluated separately. The infusion line outlet was connected to a dynamic particle counter. The antibiotic concentration was measured in an HPLC-UV assay. Result: The use of an in-line filter and an alternative saline administration route did not significantly reduce the particulate load caused by vancomycin-piperacillin/tazobactam incompatibility. Dilution of the vancomycin solution was associated with a significantly lower particulate load and maintenance of the vancomycin mass flow rate. Discussion: It is important to systematically compare the efficacy of strategies for preventing drug incompatibility. The use of diluted vancomycin solution gave the best results in the case of vancomycin-piperacillin/tazobactam incompatibility.

## 1. Introduction

Drug incompatibility is a major challenge in patients infused with several drugs—particularly in intensive care units [1,2,3]. These physicochemical incompatibilities mainly manifest themselves through the formation of a visible precipitate. However, a visible precipitate may be associated with a non-visible particulate load [4]. Drug incompatibilities have an impact on patients [5]. Precipitates can block the infusion lines, and particles infused into patients may trigger various clinical phenomena such as thrombosis, phlebitis [6,7], respiratory distress syndromes, and organ dysfunction [8,9]. It is, therefore, crucial to seek to prevent the occurrence of incompatibility during the simultaneous infusion of several drugs.

Combining two antibiotics (such as vancomycin and piperacillin/tazobactam) may lead to drug incompatibility. These two antibiotics are among the most frequently prescribed treatments in intensive care units [10,11,12]. The vancomycin-piperacillin/tazobactam (VPT) combination is recommended in several clinical guidelines as the empirical first-line treatment for a number of serious infections [13,14,15,16]. However, a white precipitate may form when the two antibiotics come into contact [17,18,19,20]. Furthermore, a large number of studies have documented the elevated risk of nephrotoxicity associated with the VPT combination [21,22,23].

In recent years, the problem of drug incompatibility has been addressed through the need to make infusion lines as safe as possible. Various strategies have been described in the literature [24]. They combine standard operating procedures with tools to counter particle and drug precipitate formation and thus the potentially associated clinical adverse events. Firstly, medical staff can use double-entry cross-tables to obtain a quick answer on the compatibility of two drugs [25,26,27,28]. The best way of avoiding drug incompatibility during an infusion is to avoid contact between the two or more incompatible drugs. Although the use of several separate infusion lines is possible, this strategy is limited by the small number of venous accesses. The sequential infusion of two incompatible drugs (interspersed by flushing of the infusion line) is only possible if neither is infused continuously [29,30,31,32,33,34]. For this reason, another approach involves limiting the contact between solutions and/or decreasing the consequences of this contact by (for example) in-line filtration [35,36,37], a particular infusion line geometry [18,38], minimization of the drug concentrations [38], the use of a multilumen line, and infusion devices with a low residual volume [18,38,39]. However, the application of these strategies in isolation might not be sufficient to control the risks. Hence, it might be necessary to combine strategies in a multimodal approach [38].

Although these various strategies are currently implemented in practice, their impact under standardized conditions has not previously been evaluated. We decided to evaluate combinations of techniques and to measure their impact in terms of the particulate load and drug availability. Hence, the objective of this in vitro study was to evaluate the impact of various strategies for preventing incompatibility between vancomycin, on one hand, and piperacillin/tazobactam on the other.

## 2. Materials and Methods

### 2.1. Experiments, Devices and Drugs

#### Infusion Line and Standard Operating Procedure

**Figure 1:**
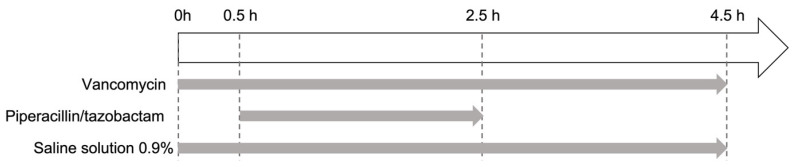
Timeline for the standard infusion of vancomycin and piperacillin/tazobactam.

The standard protocol replicated the combined infusion of vancomycin and piperacillin/tazobactam, with concomitant saline infusion over a 4.5 h period (Table 1 and Figure 1). The protocol’s flow rates and concentrations of vancomycin (4 mL/h; 20.8 mg/mL) and piperacillin/tazobactam (12.5 mL/h; 80/10 mg/mL) were those used typically in intensive care units in France (Table 1) [18].

**Figure 2:**
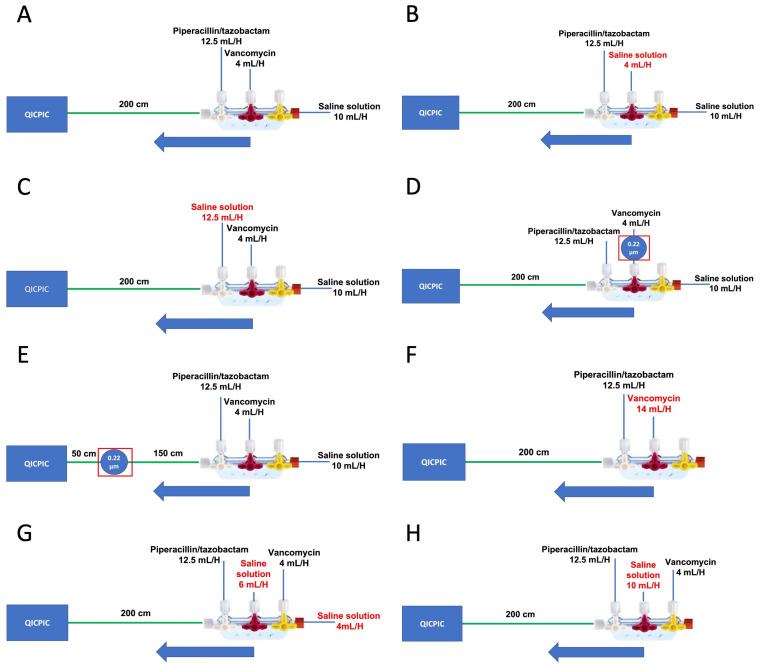
The standard manifold infusion set (**A**), the vancomycin solution replaced by SS (**B**), the piperacillin/tazobactam solution replaced by SS (**C**), the manifold infusion set with a filter on the vancomycin solution tubing (**D**), the manifold infusion set with a filter on the tubing downstream of the tap manifold (**E**), the manifold infusion set with diluted vancomycin (**F**), the manifold infusion set with two SS administration routes (**G**), and the manifold infusion set with an SS alternative administration route (**H**).

Vancomycin and saline solution (SS) were infused continuously for 4.5 h. Piperacillin/tazobactam solution was infused for 2 h (from t = 0.5 h to t = 2.5 h). The standard set-up (A) was composed of a two-port manifold and a 200 cm length of tubing (Figure 2A).

Two blank protocols were designed. In the first (B), the vancomycin solution was replaced by the diluent (i.e., SS, Figure 2B). In the second (C), the piperacillin/tazobactam solution was replaced by SS (Figure 2C).

Five additional infusion set-ups (D to H) were analyzed. They differed from the standard set-up with regard to the presence of a filter positioned at different points, the modalities of SS administration, and the dilution of the vancomycin (Figure 2D–H).

Two types of set-ups with a filter were assessed: the filter was placed either (i) between the vancomycin syringe and the manifold (D) (Figure 2D); or (ii) 150 cm downstream of the manifold (50 cm upstream of the end of the infusion line) (E) (Figure 2E).

We tested one infusion set (F) using a diluted vancomycin solution (5.95 mg/mL) at 14 mL/h and without additional hydration. The infusion flow rate 14 mL/h maintained the same mass flow rate (MFR) as in set-up A (Table 1 and Figure 2F).

Furthermore, we tested two infusion sets with different ways of administering the SS. In one (G), the hydration was split into a 4 mL/h infusion (mimicking the flow rate used to keep the veins open) and a 6 mL/h infusion (placed between the infusion ports of the vancomycin solution and the piperacillin/tazobactam solution) (Figure 2G). In the other set-up (H), SS at 10 mL/h was pumped between the infusion ports of the vancomycin solution and the piperacillin/tazobactam solution (Figure 2H).

Lastly, we compared two reconstitution and dilution procedures. The first (reconstitution and dilution with SS) is commonly used in hospital wards. The second (reconstitution with water for injection (WFI) and dilution with SS) is recommended in the summary of product characteristics (SmPC). The drugs and solvents used and the medical infusion devices are listed in Tables S1 and S2.

### 2.2. pH Measurements

All pH values were directly measured in the vancomycin syringes (20.8 mg/mL vancomycin) or vancomycin infusion bags (5.95 mg/mL vancomycin) after reconstitution and dilution with a calibrated pH meter (SB70P Symphony, VWR International, Singapore).

The pH was also measured at the outlet of the manifold during an infusion. In the first instance, the pH was measured from 10 min before the start of piperacillin/tazobactam infusion to 10 min after the start. In the second instance, it was measured from 5 min before the end of the piperacillin/tazobactam infusion to 20 min after the end. Six measurements were made for each sample and expressed as the mean ± standard deviation (SD).

### 2.3. Static Analysis of the Particulate Load

The Automated Parenteral Sampling System (APSS)-2000 particle counter (Particle Measuring Systems, Dourdan, France) was used to measure the particulate load in the various drug solutions under static conditions. This particle counter consisted of an SLS-1000 syringe containing the sample, a Liquilaz E20P light obscuration spectrometer, and a Sampler Sight-Pharma operating software (V. 3.0 SP2, Particle Measuring Systems, Dourdan, France). This apparatus meets the requirements of the European Pharmacopeia (EP) Commission. The APSS-2000 can measure particles of between 1.5 µm and 125 µm in size.

Three previously prepared syringes (20.8 mg/mL vancomycin) or infusion bags (5.95 mg/mL vancomycin) were analyzed. Four 6 mL samples were taken from each syringe or bag. The first sample was rejected. According to EP monograph 2.9.19, the loads of particles ≥10 µm in size and ≥25 µm in size should not exceed 6000 and 600 per container, respectively, for a 50 mL syringe (volume ≤ 100 mL). For an infusion bag (volume > 100 mL), the particulate load should not exceed 25/mL (particles ≥ 10 µm) or 3/mL (particles ≥ 25 µm). The results were expressed as the mean ± SD.

### 2.4. Dynamic Analysis of the Particulate Load

We used a combination of the Qicpic dynamic image analysis device (Sympatec GmbH Inc., Clausthal-Zellerfeld, Germany) with a Lixell module (Sympatec GmbH, Clausthal-Zellerfeld, Germany). The frame rate was 10 Hz and was synchronized with a high-speed camera that captured up to 500 images per second at 1024 × 1024 pixels. Using with Windox 5.0 software, we determined particle sizes between 1 µm and 30 mm and provided dynamic particle counts. The apparatus was connected to the Lixell module via Luer locks. In this study, the outlet tubing of the IV administration set was directly connected to the Qicpic, in order to obtain an accurate measurement of the particulate load every five minutes throughout the infusion. The counts of total particles, particles ≥10 µm, and particles ≥25 µm were analyzed visually (as box-and-whisker plots) and statistically. We also determined changes in the particulate load as a function of the infusion time (Q (t)).

### 2.5. Static Analysis of the Particulate Load

The drugs were assayed with an HPLC-UV method developed in our laboratory [18]. First, the compounds were separated on a reverse-phase C18 analytical column (Gemini^®^ 150 × 4.6 mm, 5 μm particle size, Phenomenex, Le Pecq, France) in gradient elution mode. The mobile phase was composed of 40 mM phosphate buffer from Supelco (EMSURE^®^ISO, 1.04873.1000, Merck, Darmstadt, Germany) in ultrapure water and adjusted to pH 5 with NaOH (A), and acetonitrile (B) (HiperSolv chromanorm for HPLC, VWR Chemicals, Fontenay-sous- Bois, France). The injection volume was 5 μL, and the flow rate was set to 1.8 mL/min. The oven was maintained at 40 °C, and the autosampler temperature was set to 20 °C. The detection wavelengths were set to 225 nm, 245 nm, and 254 nm for tazobactam sodium, vancomycin hydrochloride, and piperacillin sodium, respectively. Data were acquired using Labsolutions^®^ software (Shimadzu, Marne-la-Vallée, France).

As described by Lovich et al., the mean ± SD drug % MFR was calculated using (Equation (1) [40]):

(1) $$\%\;\mathrm{durg}\;\mathrm{MFR}=\frac{Coutlet\times Qtotal}{Csyringe\times Qsyringepump}$$

where Coutlet (mg/mL) is the concentration measured at the outlet of the infusion set, Qtotal is the total drug MFR over time (mL/h), Qsyringe pump is the drug MFR at the syringe or the volumetric pump (mL/h), and Csyringe (mg/mL) is the initial drug concentration after preparation but before infusion.

The minimum theoretical washout time (Δt (h)) for the common volume line (in mL) was defined according to the plug-flow model (Equation (2) [40]):

(2) $$\Delta t=\frac{Common\;volume}{Qtotal}$$

Using the % of vancomycin, piperacillin, and tazobactam MFR, the area under the curve (AUC) (%.h) was calculated according to the trapezoidal rule for the following four periods: 0–4.5 h, 0–0.5 h, 0.5–2.5 h, and 2.5–4.5 h.

### 2.6. Static Analysis

Particle count data were presented in box-and-whisker plots and as the mean ± SD in tables. pH data were plotted as graphs showing the mean ± SD. For HPLC-UV drug assays, the results were first plotted as the mean ± SD % drug MFR as a function of the infusion time and then expressed as the area under the curve (AUC) for the % drug (MFR) by infusion period. All data were plotted and compared using GraphPad Prism 6 Software (GraphPad Software LLC, San Diego, CA, USA) and two-tailed, non-parametric Mann-Whitney tests. The threshold for statistical significance was set to *p* < 0.05.

## 3. Results

### 3.1. The Standard Set-Up

#### 3.1.1. The Visible Particulate Load

**Figure 3:**
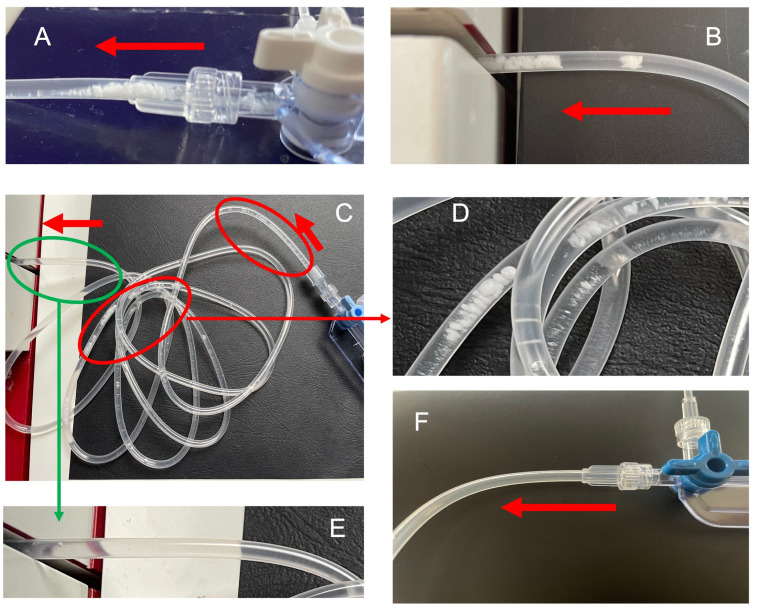
Visual observation of the infusion lines. The red arrows indicate the direction of infusion. (**A**) Formation of the initial white precipitate, following contact between the vancomycin solution and the piperacillin/tazobactam solution. (**B**) The initial precipitate is only visible at the end of the infusion tubing. (**C**) The white precipitate dissolves along the tubing during VPT co-perfusion. The red circles (**D**) correspond to the presence of visible particles, and the green circle (**E**) corresponds to the absence of visible particles. (**F**) The absence of visible precipitate during a VPT co-infusion.

**Figure 4:**
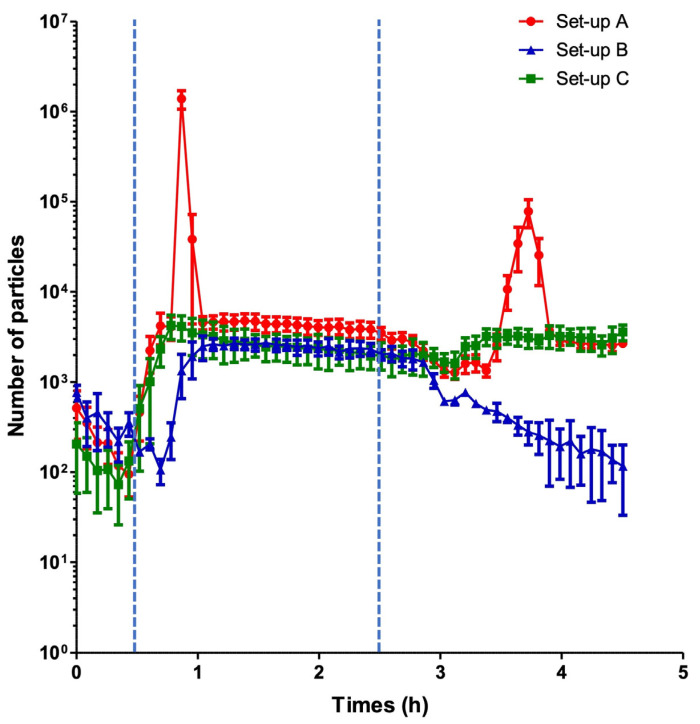
The particulate load observed during the infusion of vancomycin and/or piperacillin/tazobactam with a 200 cm manifold + extension set. The standard infusion protocol (A) is shown in red, the vancomycin-only infusion (C) is shown in green, and the piperacillin/tazobactam-only infusion is shown in blue (B). The blue dotted lines correspond to the start and the end of the piperacillin/tazobactam infusion (t = 30 min and t = 2.5 h, respectively). The results are expressed as the mean ± SD (*n* = 3–6).

From the onset of simultaneous infusions, VPT incompatibility manifested itself as a visible white, flake-like precipitate that formed at the meeting point and then migrated progressively along the tubing (Figure 3A). This precipitate migrated to the Qicpic (Figure 3B) and gave the first characteristic peak of VPT incompatibility (Figure 4).

The precipitate was initially visible at the exit of the manifold, then migrated, appeared to dissolve, and had disappeared before entering the Qicpic (Figure 3C–E). No other visible particle aggregates were present.

#### 3.1.2. The Nonvisible Particulate Load

Two particulate peaks were clearly identified during the infusion protocol. The first appeared before t = 1 h, i.e., about 30 min after the start of the piperacillin/tazobactam solution infusion. The second (smaller) peak appeared before t = 4 h; i.e., more than 1 h after stopping the piperacillin/tazobactam solution (Figure 4).

When one of the two drugs was replaced by its reconstitution solvent/diluent (set-ups B and C), no peaks were observed (Figure 4).

Between the two peaks, the particulate load was constant; these particles corresponded to those initially present in the vancomycin and piperacillin/tazobactam solutions. There was no significant difference between set-up A and set-ups B + C with regard to the particulate load present between the two peaks during the same time interval (respectively, 91,814 ± 36,074 vs. 122,012 ± 16,856; *p* = 0.2619, Mann-Whitney, *n* = 3–6).

In set-up A, 95.3% of the particles ≥ 10 µm and 99.8% of the particles ≥ 25 µm were found in peaks 1 + 2 (Table 2).

### 3.2. The Influence of In-Line Filters

#### 3.2.1. Placement of an In-Line Filter on the Infusion Line of the Vancomycin Solution (Set-Up D)

**Figure 5:**
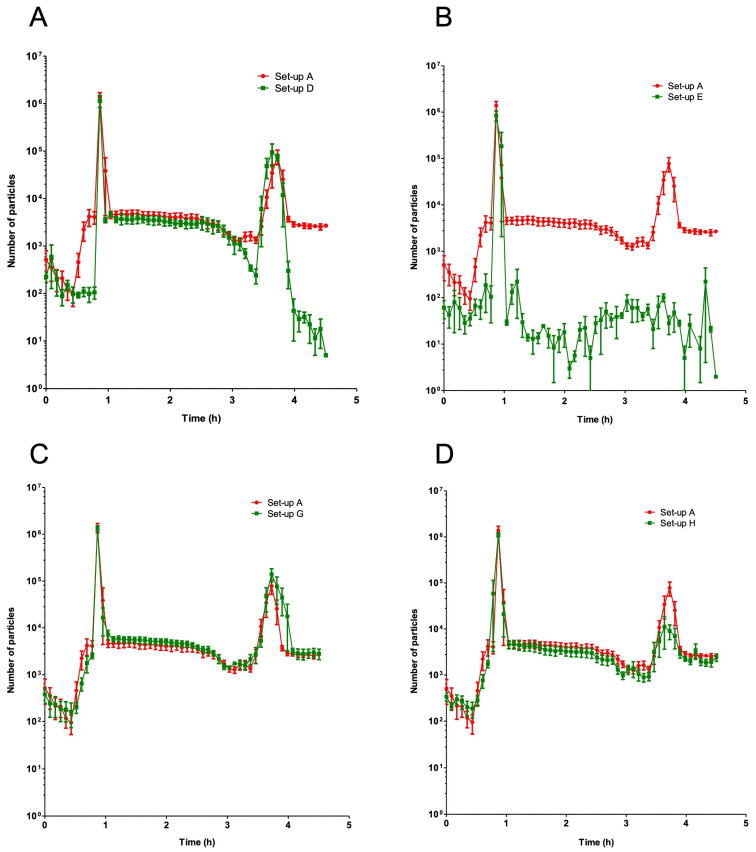
The particle distribution over time, showing the impact of in-line filtration and the hydration position on the particulate load in set-up D (**A**), set-up E (**B**), set-up G (**C**), and set-up H (**D**). The results are expressed as the mean ± SD (*n* = 5 or 6).

The use of a filter on the vancomycin solution’s infusion line (set-up D) gave the same visual observation results as the standard infusion set (set-up A) (Figure 3A–C). Furthermore, the filter did not reduce the two particle peaks characteristic of VPT incompatibility (Figure 5A). The particulate load was lower during the periods when vancomycin solution and hydration solution were infused (t = 0.5 h −> t = 0.83 h, and t = 3h −> t = 4.5 h (excluding the second peak)).

**Figure 6:**
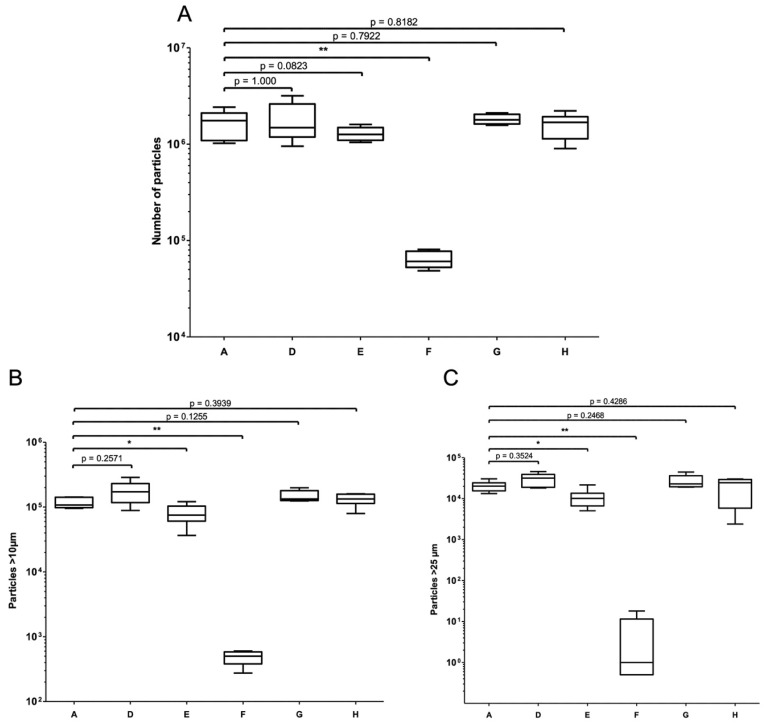
Impact of the choice of infusion set or protocol on the particulate load. Comparisons of the total particulate load (**A**), the particulate load ≥10 µm (**B**), and the particulate load ≥25 µm (**C**) in the various infusion sets and protocols (set-ups A, D, E, F, G, and H). The results are expressed as the median (range) (* *p* < 0.05 and ** *p* < 0.01 in a Mann-Whitney test, *n* = 5–6).

For set-up D, 98.9% of particles ≥10 µm and 99.9% of particles ≥25 µm were found in the pooled peaks 1 and 2. The standard infusion set (set-up A) and set-up D did not differ significantly in the loads of total particles, particles ≥10 µm and ≥25 µm (respectively, 1,679,849 ± 544,761 vs. 1,915,989 ± 448,388; *p* = 1.000; 115,494 ± 21,315 vs. 173,237 ± 39,615 *p* = 0.2571; 20,469 ± 6023 vs. 25,421 ± 12,921; *p* = 0.3524) (Figure 6 and Table 2).

#### 3.2.2. In-Line Filter Downstream of the Manifold (Set-Up E)

No visible aggregates were observed downstream of the filter (Figure 3E).

The use of in-line filters reduced the particulate load over the whole infusion and made the second particle peak disappear. The first particle peak persisted, despite the presence of the filter (Figure 5B). For this set-up, 99.9% of particles ≥10 µm and 99.9% of particles ≥25 µm were found in the first (sole) peak (Table 2).

Set-up A and set-up E did not differ significantly in the total particulate load (1,679,849 ± 544,761 vs. 927,494 ± 301,667; *p* = 0.0823), although the loads of particles ≥10 µm and ≥25 µm were significantly lower in set-up E than in set-up A (respectively, 53,937 ± 16,749 vs. 115,494 ± 21,315; *p* < 0.05 and 6069 ± 3149 vs. 20,469 ± 6023; *p* < 0.05) (Figure 6 and Table 2).

### 3.3. The Influence of Alternative SS Administration Routes (Set-Ups G and H)

The use of alternative SS administration routes (set-ups G and H) gave the same visual observation results as the standard infusion set (set-up A) (Figure 3A–C). Regardless of the changes in the hydration positions, the two characteristic particle peaks of VPT incompatibility remained present (Figure 5C,D).

For set-up G, 97.6% of the particles ≥10 µm and 99.8% of the particles ≥25 µm were found in the compilations of peaks 1 and 2. The standard set-up and the set-up G did not differ significantly in the loads of total particles, particles ≥10 µm, and particles ≥25 µm (1,679,849 ± 544,761 vs. 1,823,449 ± 219,617; *p* = 0.7922; 115,494 ± 21,315 vs. 148,694 ± 31,198; *p* = 0.1255; 20,469 ± 6023 vs. 26,848 ± 10,570; *p* = 0.2468, respectively) (Figure 6 and Table 2).

For the set-up H, 97.3% of particles ≥10 µm and 99.6% of particles ≥25 µm were found in peaks 1 + 2. The standard set-up and the set-up H did not differ significantly in terms of the loads of total particles, particles ≥10 µm, and particles ≥25 µm (respectively, 1,679,849 ± 544,761 vs. 1,727,053 ± 363,369; *p* = 0.8182; 115,494 ± 21,315 vs. 132,092 ± 32,742; *p* = 0.0.3939; 20,469 ± 6023 vs. 17,503 ± 9773; *p* = 0.4286, respectively) (Figure 6 and Table 2).

### 3.4. Impact of Dilution of the Vancomycin Solution (Set-Up F)

#### 3.4.1. The pH and the Particulate Load in the Diluted Vancomycin Solution

Dilution of the vancomycin solution resulted in a slight increase in the pH (3.39 to 3.66) (Table 3).

After reconstitution with SS, the 20.8 mg/mL vancomycin solution (volume ≤ 100mL) and the 5.95 mg/mL vancomycin solution (volume > 100 mL) did not comply with the EP specifications for the particulate load ≥ 10 µm (<6000 particles/container for standard solutions and < 25 particles/mL for diluted solutions, as mentioned above) (Table 4).

After reconstitution with WFI (based on vancomycin’s SmPC), the standard solution (volume ≤ 100 mL) and the diluted solution (volume > 100 mL) complied with the EP specifications for the particulate load ≥ 10 µm.

#### 3.4.2. The pH and the Particulate Load in the Infusion, and Drug Assays

No visible particles were observed at the point where the diluted vancomycin solution and the piperacillin solution met (Figure 3F).

- pH

**Figure 7:**
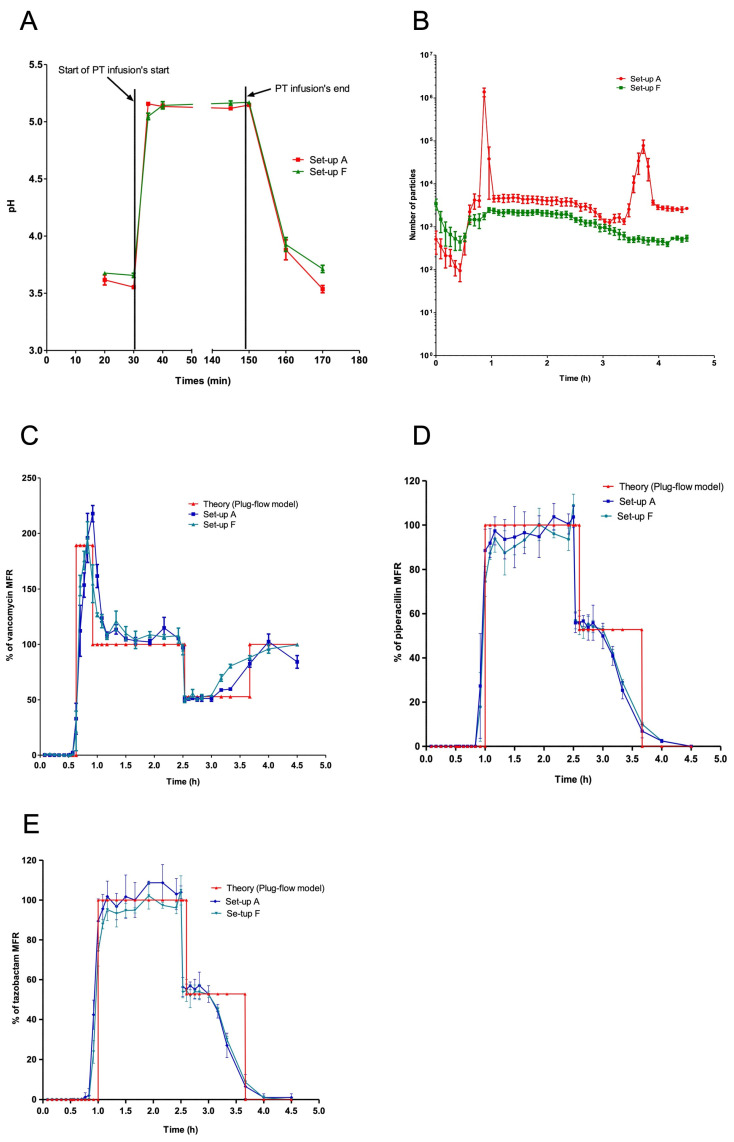
Influence of the vancomycin solution concentration on the specific charge, solution pH and active ingredient MFR. (**A**) Change in pH during VPT co-infusion with a 20.8 mg/mL or 5.95 mg/mL vancomycin solution. The results are expressed as the mean ± SD, *n* = 3. (**B**) The particulate load as a function of the infusion time for set-up A (in red) and set-up F (in green). The results are expressed as the mean ± SD, *n* = 6. (**C**–**E**) Change over time in the experimental/theoretical MFR (%) in the plug-flow model of vancomycin (**C**), piperacillin (**D**), and tazobactam (**E**) at the manifold and in the tubing infusion line in set-up A or set-up F. The results are expressed as the mean ± SD, *n* = 3.

Before infusion of the PT solution, the mean pH at the outlet of the manifold was similar in set-ups A and F (3.59 ± 0.06 vs. 3.67 ± 0.03, respectively; *p* = 0.0534). An increase in pH was observed when PT infusion was initiated. During co-perfusion, the pH did not change in either set-up, and the values remained similar (5.14 ± 0.03 vs. 5.13 ± 0.06 for set-ups A and F, respectively; *p* = 0.7494). A decrease in pH was observed when the piperacillin/tazobactam infusion was stopped (3.71 ± 0.21 vs. 3.82 ± 0.14 for set-ups A and F, respectively; *p* = 0.2403). The decrease was similar in the two set-ups (Figure 7A).

- Particles

During infusion of the 5.95 mg/mL vancomycin solution, no particulate peaks were observed at the manifold or in the tubing (Figure 7B). A significant higher load of total particles, particles ≥ 10 µm and particles ≥ 25 µm was observed in set-up A (containing 20.8 mg/mL vancomycin), relative to set-up F (containing 5.95 mg/mL vancomycin), with values of 1,679,849 ± 544,761 vs. 64,300 ± 13,162 (*p* < 0.01), 115,494 ± 21,315 vs. 485± 127 (*p* < 0.01), and 20,469 ± 6023 vs. 5 ± 8 (*p* < 0.01), respectively (Figure 6 and Table 2).

- Drug assays

In Mann-Whitney tests (*n* = 3), there were no significance differences in drug mass flows between set-ups A and F. The vancomycin experimental/theoretical mass flow % did not vary significantly as a function of the infusion period in either set-up (Table 5, Figure 7C). The same was true for the % MFR of the piperacillin and tazobactam solutions (Table 6 and Table 7, Figure 7D,E).

## 4. Discussion

In an in vitro study, we evaluated the influence of various strategies used in healthcare facilities to limit the occurrence of drug incompatibilities. We focused on the simultaneous infusion of VPT and the well-known incompatibility between the two components [18,19,41,42]. Our experiments highlighted the influence of vancomycin dilution on the particulate load generated by VPT drug incompatibility.

### 4.1. Impact of Solvent Reconstitution

The first point to emphasize is the importance of choosing the right solvent for reconstituting drugs for infusion. Indeed, the reconstitution/dilution stage is crucial for appropriate management of the patient’s medication. According to the SmPC for vancomycin, WFI is recommended for reconstitution, followed by SS for dilution [43]. According to the literature, the particulate load and/or the vancomycin solution concentration are not compliant when vancomycin is not reconstituted with WFI [18,44,45]. Our results confirmed this: only vancomycin reconstituted with WFI meets EP standards [46].

Although reconstitution with WFI will not alone avoid the occurrence of drug incompatibility, this observation confirms the importance of following the reconstitution/dilution guidelines, reducing the particulate load initially present in infused drug solutions, and thus avoid the administration of particulate matter to patients.

### 4.2. VPT Incompatibility

In a visual analysis, drug incompatibility instantly produces a white precipitate at the point where the vancomycin solution and the piperacillin/tazobactam solution meet. Although a precipitate is visible all along the tubing at the very start of the co-infusion, it eventually disappears. These results are in line with Nichols et al.’s report of a visible, milky precipitate that appeared during the initial mixing and eventually disappeared upon agitation [47]. These observations suggest that the precipitate dissolves over time in the tubing. Kufel et al. did not observe any visible precipitate during tests simulating the Y-site, whereas a precipitate was observed during an actual Y-infusion [17].

With regard to nonvisible particles, the delay in the appearance of the peaks appears to be related to the length of the tubing (200 cm; dead volume: 10 mL) and the flow rate. Almost all the particles—more than 95% of those ≥ 10 µm in size and more than 99% of those ≥ 25 µm—are found in the two peaks. The formation of these particles might be related (at least in part) to changes in flow rates at the start and end of the piperacillin/tazobactam infusion. Between the two peaks, the particulate count was similar to those measured in “blank” tests (set-ups B and C) and was derived from the particles already present in the infusion solutions. This is related to the fact that no visible precipitate was observed at the end of the tubing during the incompatibility.

VPT incompatibility does not appear to be an acid-base phenomenon. Indeed, the pH variations were similar in the infusions with standard and diluted vancomycin solutions. Importantly, a visible precipitate was observed in the standard vancomycin protocol, but not in the diluted vancomycin protocol. Given that the pH and the vancomycin MRF were very similar, we cannot readily explain the absence of a precipitate in the “diluted vancomycin” protocol. It is known that vancomycin dimerizes at pH = 5 and 25 °C [48,49], and so it remains to be determined whether this dimerization is involved (at least in part) in the presence or absence of a precipitate. Although precipitate formation during VPT incompatibility is well documented, the precise mechanisms and composition are not fully understood and require more research.

### 4.3. Value of In-Line Filters in VPT Incompatibility

In vitro studies have clearly shown the impact of filters on the retention of particles present in infused solutions [50,51]. In the present in vitro study, no precipitate was observed downstream of the in-line filter.

The clinical impact of in-line filters is subject to debate. Some studies have clearly shown that filters reduce the occurrence of complications in pediatric or adult populations [37,52]. Other studies did not find any differences between patient groups with vs. without in-line filters [53,54,55]. These studies did not provide details of what was administered to the patients and whether drug incompatibility was present during the infusion protocols. This lack of detail might explain the observed discrepancies.

Very few in vitro or clinical studies have evaluated the effectiveness of a filter during drug incompatibility [51]. Our present results showed that in-line filters were effective but could not produce particle-free solutions. On the same lines, Masse et al. reported that particles ≥ 10 µm and ≥25 µm in size were present in filtered vancomycin solutions [45]. Our results highlight a major decrease in the particulate load and the disappearance of the second peak after filtration. However, the first particulate peak—the one containing the majority of the particulate load—was still present. Re-precipitation is a possible explanation for maintenance of the first peak. However, this goes against the dissolution of the precipitate observed in the experiments described above. At present, we cannot explain the maintenance of the first peak. No visible precipitate was observed downstream of the filter. The filter appeared to be intact, and tests on another drug incompatibility showed that the filter was still functional. Further work to understand the origin and nature of this particulate load despite the presence of a filter is necessary.

The persistence of the peak despite the presence of an in-line filter is specific to VPT incompatibility. The filter remains an effective barrier to other incompatibilities, such as the mixture of a dobutamine solution with a furosemide solution. Our results confirm that each drug incompatibility has particular features, and indicated that each incompatibility must be studied and dealt with on a case-by-case basis.

### 4.4. The Influence of Vancomycin Dilution on VPT Incompatibility

We found that the best strategy for mitigating VPT incompatibility was dilution of the vancomycin solution. This was the only strategy that significantly reduced the particulate load and removed the two particle peaks. Moreover, the mass concentrations were close to those expected.

Many in vitro studies have highlighted the value of using a vancomycin solution at a maximum concentration of 5 mg/mL [56,57,58]. In our study, the concentration used was close to the recommended value.

Clinically, it is known that infusions of vancomycin solutions are more prone to complications than other antibiotics [59]. However, the use of a concentration ≤5 mg/mL is also recommended in clinical studies, particularly when the vancomycin solution is infused via the peripheral route. Various studies have evaluated the safety of peripheral vancomycin infusion. A vancomycin solution ≤5 mg/mL is safe and might decrease the risk of complications in venous systems [60,61]. However, a recent study of a small group of patients showed that use of a 4 mg/mL vancomycin solution only delayed (but did not prevent) the occurrence of phlebitis [62]. The use of diluted vancomycin solutions obliges healthcare services to change their procedures because electric syringe pumps have to be replaced by infusion pump systems.

### 4.5. Homogenization of Solutions in Medical Devices

The results obtained here raise questions about mixing solutions in infusion lines. The MFR where the vancomycin solution met the piperacillin solution was the same for the standard vancomycin solution and the diluted vancomycin solution. However, the visual observations and particulate loads were quite different, which suggests that the vancomycin solution and the SS diluent to not mix homogeneously. The use of a diluted vancomycin solution avoids the problem of dilution in the infusion line.

The protocol could be optimized by infusing the vancomycin solution and the SS upstream of the piperacillin/tazobactam solution; the common volume before the encounter with the piperacillin/tazobactam solution would be larger.

## 5. Conclusions

The efficacy of these strategies for dealing with incompatibility varies according to the drugs used, the concentration, the infusion rate, and other physicochemical conditions. It is important to always evaluate the efficacy of a given strategy on known drug incompatibilities. In the particular case of VPT incompatibility, the infusion of a dilute (~5 mg/mL) vancomycin solution appears to give the best results for the particulate load while maintaining the % experimental/theoretical MFR.

## Figures and Tables

**Table 1:** In vitro preparation of the drugs in the standard protocol and in the diluted protocol.

Drug or Injectable Product	Set-Ups	Reconstitution/Dilution (0.9% SS)	Container	Concentration (mg/mL)	Infusion Flow Rate (mL/h)
Vancomycin	A, B, C, D, E, G, H	48 mL q.s.	Syringe	20.8	4
	F	168 mL q.s.	Infusion bag	5.95	14
Piperacillin/ tazobactam	A, B, C, D, E, F, G, H	50 mL q.s.	Syringe	80/10	12.5
0.9% saline solution	A, B, C, D, E, H	250 mL q.s.	Infusion bag	-	10
	F	-	-	-	-
	G	50 mL q.s.	Syringe	-	6
		250 mL q.s.	Infusion bag	-	4

**Table 2:** Total particulate load and loads of particles ≥10 µm and particles ≥25 µm throughout the infusion, at the time of peak 1, at the time of peak 2, and at times of peaks 1 and 2 for set-ups A to H. The results are expressed as the mean ± SD (*n* = 5–6). The particulate load in a given period is also expressed as percentage of the load over the total infusion period.

	Total Infusion Time			Peak 1			Peak 2			Peak 1 + 2		
Particles	Total number	≥10 µm	≥25 µm	Total number	≥10 µm	≥25 µm	Total number	≥10 µm	≥25 µm	Total number	≥10 µm	≥25 µm
Set-up A	1,679,849 ± 544,761	115,494 ± 21,315	20,469 ± 6023	84.2% (1,450,728)	86.6% (101,146)	94.0% (19,266)	8.7% (186,854)	8.7% (14,065)	5.7% (1704)	92.9% (1,575,297)	95.3% (110,523)	99.8% (20,402)
Set-up B	66,310 ± 14,182	1343 ± 910	16 ± 19	no peak			no peak			no peak		
Set-up C	122,123 ± 59,907	4052 ± 1723	32 ± 30	no peak			no peak			no peak		
Set-up D	1,915,989 ± 448,388	173,237 ± 39,615	25,421± 12,921	66.7% (1,160,500)	69.9% (109,329)	86.9% (21,927)	28.31% (687,548)	29% (62,320)	13.03% (3481)	95.0% (1,478,438)	98.9% (137,318)	99.9% (20,326)
Set-up E	927,494 ± 301,667	53,937 ± 16,749	6069 ± 3149	99.7% (924,527)	99.9% (53,879)	99.9% (6065)	no peak			99.7% (924,527)	99.9% (53,879)	99.9% (6065)
Set-up F	64,300 ± 13,162	485 ± 127	5 ± 8	no peak			no peak			no peak		
Set-up G	1,823,449 ± 219,617	148,694 ± 31,198	26,848 ± 10,570	74.1% (1,360,676)	79.1% (114,999)	81.3% (20,457)	18.3% (329,369)	18.5% (30,141)	18.6% (6355)	92.5% (1,690,045)	97.6% (145,140)	99.8% (26,812)
Set-up H	1,727,053 ± 363,369	132,092 ± 32,742	17,503 ± 9773	88.5% (1,358,492)	93.6% (137,979)	98.7% (25,688)	3.0% (46,076)	3.7% (5414)	0.3% (238)	91.5% (1,404,568)	97.3% (143,393)	99.6% (25,926)

**Table 3:** pH values for the nondiluted and diluted vancomycin solutions (mean ± SD; *n* = 3).

	pH
20.8 mg/mL vancomycin solution	3.39 ± 0.02
5.95 mg/mL vancomycin solution	3.66 ± 0.02

**Table 4:** The load of particles ≥10 µm and particles ≥ 25 µm for 20.8 mg/mL and 5.95 mg/mL vancomycin solutions, according to the reconstitution/dilution method (SS/SS or WFI/SS) (mean ± SD; *n* = 9).

Vancomycin Solution	Reconstitution Solvent/Diluent	Particles ≥ 10 µm	Particles ≥ 25 µm
20.8 mg/mL	SS/SS	9524 ± 1168 (particles/container)	61 ± 39 (particles/container)
	WFI/SS	2721 ± 151 (particles/container)	30 ± 5 (particles/container)
5.95 mg/mL	SS/SS	84 ± 16 (particles/mL)	2 ± 1 (particles/mL)
	WFI/SS	12 ± 2 (particles/mL)	1 ± 1 (particles/mL)

**Table 5:** Comparison of the AUC for the experimental/theoretical vancomycin hydrochloride mass flow %.h, in a Mann-Whitney test (*n* = 3).

Vancomycin	Median ± SD AUC for Set-Up A	Median ± SD AUC for Set-Up F	*p* Value
Total infusion time (h)	359.9 ± 19.1	382.5 ± 9.9	0.7
0–0.5 h	0.02 ± 0	0.0008 ± 0	0.5
0.5–2.5 h	222.9 ± 10.7	226.8 ± 11.4	>0.9999
2.5–4.5 h	142.8 ± 10.2	156.8 ± 3.2	0.2

**Table 6:** Comparison of the AUC for the experimental/theoretical piperacillin mass flow %.h, in a Mann-Whitney test (*n* = 3).

Piperacillin	Median ± SD AUC for Set-Up A	Median ± SD AUC for Set-Up F	*p* Value
Total infusion time (h)	202.5 ± 14.1	199.5 ± 6.2	0.7
0–0.5 h	0 ± 0	0 ± 0	>0.9999
0.5–2.5 h	150.0 ± 9.3	147.7 ± 5	0.4
2.5–4.5 h	48.9 ± 5.6	48.7 ± 1.2	>0.9999

**Table 7:** Comparison of the AUC for the experimental/theoretical tazobactam mass flow % h, in a Mann-Whitney test (*n* = 3).

Tazobactam	Median ± SD AUC for Set-Up A	Median ± SD AUC for Set-Up F	*p* Value
Total infusion time (h)	211.7 ± 17.9	202.7 ± 6.8	0.7
0–0.5 h	0 ± 0	0 ± 0	>0.9999
0.5–2.5 h	156.0 ± 13.1	151.4 ± 4.7	0.4
2.5–4.5 h	49.1 ± 6.6	48.6 ± 2.1	>0.9999

## Data Availability

Not applicable.

## Supplementary Materials

The following supporting information can be downloaded at: https://www.mdpi.com/article/10.3390/pharmaceutics15082069/s1, Table S1: The infused drugs and diluents used in the in vitro study; Table S2: The medical devices used for preparation and infusion in the present in vitro study.
