# Supplementary material for: Evaluation of Strategies for Reducing Vancomycin-Piperacillin/Tazobactam Incompatibility
Source: Pharmaceutics. 2023 Aug 1;15(8):2069. doi: 10.3390/pharmaceutics15082069 (PMC10459903; doi:10.3390/pharmaceutics15082069)
Supplement: Supplementary file 1 [file pharmaceutics-15-02069-s001.zip › pharmaceutics-2509331-supplementary.pdf]

Supplementary methods

Products	Manufacturer	Initial concentration / Dose	Batch numbers	Expiration dates
**Vancomycin**	Mylan	1 g	C0312<br>C0274<br>C0323	2023-10<br>2023-09<br>2023-09
**Piperacillin/tazobactam (PT)**	Panpharma	4 g/500 mg	307484<br>306723	2025-02<br>2023-12
**Saline solution (SS)**	Baxter	0.9%	22B25T1G<br>22D18T1B<br>21L01T1D	2024-01<br>2024-01<br>2023-11

Table S1: The infused drugs and diluents used in the *in vitro* study

Medical devices	Manufacturer	References	Batch numbers	Expiration dates
**Needles 1.2 mm x 40 mm**	Becton Dickinson, Fraga, Spain	K111815<br>304622	80108010<br>1805 15	2026-08-11<br>2023-04
**Syringe 20 mL Luer-lock BD Plastipak**	BD Switzerland Sàrl, Eysins, Switzerland	300629	2102012	2026-01-31
**Syringe 50 mL Luer-lock BD Plastipak**	BD Switzerland Sàrl, Eysins, Switzerland	300865	2204025	2027-03-31
**Three-port manifold + 150 cm PE/PVC extension line**	CAIR LGL, Lissieu, France	RPB3315	22A24-TJB	2026-12-24
**PE/PVC extension line**	CAIR LGL, Lissieu, France	PB3115	22C28-TNPW	2027-02-28
**Four-port manifold + 200 cm PE/PVC extension line**	CAIR LGL, Lissieu, France	RPB4320	22C23-TWNP	2027-02-23
**Infusion pump set**	Fresenius Kabi AG, Bad Homburg, Germany	M46441000S<br>Z072810F	84461131<br>84171129	2024-11-14<br>2022-04
**Syringe pump**	Fresenius Vial, Brezins, France	083010		
**Infusion pump**	Fresenius Vial, Brezins, France	Volumat MC agilia F	EP5113-0	

Table S2: The medical devices used for preparation and infusion in the present *in vitro* study
